# Development of a decentralized cohort for studying post-acute sequelae of COVID-19 in India in the Data4life Study

**DOI:** 10.1038/s43856-023-00349-y

**Published:** 2023-08-25

**Authors:** Josh Schilling, Sepideh Shokouhi, Aisha Montgomery, Girish N. Nadkarni, Alexander W. Charney, Anil Shanker, Rajbir Singh, Kenar Jhaveri, Karandeep S. Singh, Prashant Khadke, Praduman Jain

**Affiliations:** 1 Vibrent Health, 4114 Legato Rd #900, Fairfax, VA 22033 USA; 2 Icahn School of Medicine at Mount Sinai, 1 Gustave L. Levy Pl, New York, NY 10029 USA; 3 Meharry Medical College, 1005 Dr DB Todd Jr Blvd, Nashville, TN 37208 USA; 4 Donald and Barbara Zucker School of Medicine at Hofstra/Northwell, 500 Hofstra Blvd, Hempstead, NY 11549 USA; 5 Learning Health Sciences, University of Michigan, 1111 E Catherine St, Ann Arbor, MI 48109 USA; 6 Pensieve Health, 3A, 3rd Floor, Vascon Wekfield Chambers, Satpal Malhotra Marg, Nagar Road, Pune, Maharashtra 411014 India

**Keywords:** Medical research, Public health

## Abstract

**Background:**

Decentralized, digital health studies can provide real-world evidence of the lasting effects of COVID-19 on physical, socioeconomic, psychological, and social determinant factors of health in India. Existing research cohorts, however, are small and were not designed for longitudinal collection of comprehensive data from India’s diverse population. Data4Life is a nationwide, digitally enabled, health research initiative to examine the post-acute sequelae of COVID-19 across individuals, communities, and regions. Data4Life seeks to build an ethnically and geographically diverse population of at least 100,000 participants in India.

**Methods:**

Here we discuss the feasibility of developing a completely decentralized COVID-19 cohort in India through qualitative analysis of data collection procedures, participant characteristics, participant perspectives on recruitment and reported study motivation.

**Results:**

As of June 13th, 2022, more than 6,000 participants from 17 Indian states completed baseline surveys. Friend and family referral were identified as the most common recruitment method (64.8%) across all demographic groups. Helping family and friends was the primary reason reported for joining the study (61.5%).

**Conclusions:**

Preliminary findings support the use of digital technology for rapid enrollment and data collection to develop large health research cohorts in India. This demonstrates the potential for expansion of digitally enabled health research in India. These findings also outline the value of person-to-person recruitment strategies when conducting digital health research in modern-day India. Qualitative analysis reveals opportunities to increase diversity and retention in real time. It also informs strategies for improving participant experiences in the current Data4Life initiative and future studies.

## Plain language summary

Due to the vast geographical size and ethnic diversity of the population, India represents a huge challenge for conducting research studies. The Data4Life study was set up to understand if digital tools can be an effective way to study long-term effects of COVID-19 across India. We studied different ways of collecting the relevant information from participants, the background of each participant, reasons, and motivation of each participant for joining the study. The results showed that friend and family referrals were the most common recruitment reason. Helping family and friends was reported as the main motivation for joining the study. Overall, the findings support the use of digital tools as an effective recruitment method for research studies in India.

## Abstract (web summary)

Schilling et al. devise a large-scale decentralized cohort to study the post-acute sequelae of COVID-19 in India. Preliminary findings support the use of digital technology to enable health research studies and highlight some improvement opportunities for further expansion of the cohort.

## Introduction

The Coronavirus disease 2019 (COVID-19) has led to a dramatic health crisis in India, the second most populated country in the world^1^. Early efforts to mitigate the spread of the infection focused on quarantine orders, workplace closures, and social distancing practices that began with India’s national lockdown^2^. Although initially declared for 21 days on March 25, 2020, the lockdown was later extended for 55 days to reduce virus transmission. These lifesaving actions came with socioeconomic consequences^3^ that continue to disrupt the daily lives of Indians in unprecedented ways^4^. Loss of income, limited access to healthcare, and rising rates of behavioral health symptoms are among the known secondary effects of COVID-19^5–7^. Research shows a disproportionate impact of the pandemic across India’s population among low-income households^2,8^, women^9–11^, and patients with pre-existing chronic conditions^12^. Younger populations are also shown to be more susceptible to mental health problems due to COVID-related disruptions in recreational and educational activities^13^. Currently, more data is needed to capture the long-term, compound effects of COVID-19 in India. Digital health technologies can effectively capture real-world data to better classify the wide range of physical, psychological, cultural, social, and economic effects of post-COVID life.

The development of digitally enabled cohorts presents more challenges in India than in Western countries^14–16^, leading to a scarcity of national research-grade digital health data. These challenges include low digital literacy, a shortage of skilled workforce in health informatics, and a lack of data governance and protection policies. India’s healthcare sectors have leveraged some digital tools to combat the pandemic, however, they are primarily used for COVID-19 contact tracing, infection risk mitigation, and tracking vaccination rates^17^. Existing survey tools have allowed researchers to gather episodic, post-COVID health information from small population samples across India^7,18^. Unfortunately, the tools currently in use are not designed for longitudinal studies requiring secure, multidimensional health data collection and participant engagement from large populations across India.

The Data4Life Consortium was launched in March 2022 as India’s initial digitally enabled, nationwide health cohort to study how individuals, families, communities, and regions were affected by lasting post-COVID conditions and post-acute sequelae of COVID-19 (PASC). The Consortium was a public-private collaboration between academic institutions in the United States, Health Centers in India, and industry partners in digital health technology. The Consortium established a secure, participant-centered digital platform to facilitate participant engagement, remote electronic consent, and collection of longitudinal health information for this study. Data4Life aimed to create a unique data repository for researchers to assess differences in socioeconomic factors, lifestyle, and health contributing to long-term COVID-related outcomes throughout India and to advance infectious disease knowledge.

Data4Life sought to use digital recruitment and data collection methods to investigate the long-term effects of COVID-19 in India, one of the most genetically and ethnically diverse populations in the world^19^. The study aimed to enroll at least 10,000 participants in the first 12 months. This report provides a detailed description of the study framework and procedures along with demographic characteristics and preliminary findings amongst participants recruited in the first three months of the study. The current discussion will examine the recruitment preferences and study motivation of currently enrolled participants to identify factors contributing to effective digital recruitment in this population. This review will provide real-time insights into the successes and challenges in achieving cohort diversity and identify potential areas requiring further development in the ongoing study. Preliminary findings will inform future strategies for increasing diversity and improving participant experiences in Data4Life and other large, international digitally enabled health studies.

As of June 13th, 2022, the study has enrolled over 6000 participants from 17 Indian states, with friend and family referrals being the most common recruitment method (64.8%) across several demographic groups. Joining the study to support family and friends remains the primary motivation (61.5%). These findings support the utilization of digital technology for swift enrollment and data collection in large health research cohorts in India, highlighting the potential for further expansion. Person-to-person recruitment strategies prove valuable in modern-day Indian digital health research. The qualitative analysis identifies real-time opportunities to enhance diversity, retention, and improve participant experiences in Data4Life and future studies.

## Methods

### Study Design

Data4Life was an observational, longitudinal study with an unrestricted sample size. The 12-month pilot phase aimed to enroll 10,000 + COVID-positive individuals across diverse communities in India and follow them for one year through monthly data collection. The study was launched collaboratively between a US-based digital health technology provider, Vibrent Health, and several clinical research centers in India: Pensieve Health (Mumbai), Hande Hospital (Panvel, Navi Mumbai), ACUMDX Laboratory and Research Center (Ghatkopar, Mumbai, Kota and Jodhpur in Rajasthan), and Sun Diagnostics (Ghatkopar, Mumbai). All centers were approved to conduct SARS-COVID-19 testing during the pandemic. Study activities were conducted according to the US Code of Federal Regulations Title part 46 and approved by the Royal Pune Independent Ethics Committee in India. Local researchers in India were involved with the study design, implementation, intellectual property, and authorship of publications. Eligible participants were identified in databases of over 20,000 patients who previously tested COVID-positive at an affiliated hospital or laboratory. Clinical coordinators contacted eligible individuals and members of their households by email or phone call. Data4Life participants had the option to invite family members and friends to join the study. Social media and email campaigns were not used for active recruitment; however, enrolled participants were able to send email invitations to their social networks. Furthermore, as the pandemic progressed, increased COVID testing resulted in increased promotion of the Data4life study among friends, families, and neighbors. Study recruitment began on March 15, 2022, and continued through August 03, 2022. All participants provided informed consent.

Data4Life participation was open to all English-literate residents in India, age 18+ years, with a history of having tested COVID-positive. The study recruitment website (www.data4lifestudy.org) addressed study aims, eligibility criteria, data privacy, and terms of service and directly linked participants to a mobile application store to download the study app. Participants were required to register for an account via the web or study app to complete electronic consent (eConsent) and enroll in the study. Participants used the web or mobile Data4Life study app to complete all survey modules. Optional follow-up surveys were deployed every four weeks for 12 months after baseline. The rationale for choosing monthly surveys was based upon rapid changes in the COVID landscape and the anticipated emergence of new variants^20^. To establish trust, participants could easily withdraw from the study at any time through the Data4Life study app and opt out of all future correspondence. Participants had access to study-related educational content and could complete optional community-based quick polls for continued study engagement. Further, ongoing study insights were shared publicly via the website and mobile app and will be published in scientific journals to promote transparency.

### Post-acute sequelae of COVID-19 (PASC)

The World Health Organization (WHO) defines the post-acute COVID-19 condition (PASC), also known as long COVID, as a long-term continuance of symptoms following an acute COVID-19 infection^21^. This definition also includes the development of symptoms after initial recovery from acute COVID infection. Data4Life adopts the definition of PASC provided by the US Center for Disease Control (CDC): the continuance, recurrence, or presentation of COVID-19 symptoms four (4) or more weeks after the initial COVID-19-positive test as self-reported by participants^22^. Symptoms occurring in association with an initial COVID-19-positive infection, or re-infection, four or more weeks after an initial COVID-19-positive confirmed test were not classified as long-COVID for the purpose of this study. Due to the changing landscape of the pandemic, a wide range of symptom data was collected. In previous studies, the most frequently reported long-lasting COVID-related symptoms were fatigue, shortness of breath, headache, loss of taste/smell, impaired tolerance of physical activity, and depressed mood^23,24^. For completion, Data4Life captured these along with nine (9) additional, less frequently reported symptoms: sleep disruption, poor memory or brain fog, joint pain, cough, dizziness, anxious mood, gastrointestinal symptoms, nasal symptoms, and heart palpitations. Though it has emerged as a relevant PASC symptom, our study did not adequately capture symptoms of myalgia. Previous clinic-based studies conducted in India provided limited data as data collection was limited by clinic site, and sample sizes were <1500^25–27^. Therefore, the decentralized, remote recruitment and data collection methods utilized in Data4Life were fundamental to capturing a larger, more diverse representation of PASC in India.

### Data security

The Data4Life study was conducted according to established industry-standard policies, procedures, and technology system guidelines. These included, but were not limited to, the Security and Privacy Controls for Federal Information Systems and Organizations (NIST SP 800-53 r4), the NIST Risk Management Framework (NIST SP 800-39), FIPS 199, NIST SP 800-37, the NIST Cybersecurity Framework, the Guide for Applying the Risk Management Framework to Federal Information Systems: A Security Life Cycle Approach (latest revision), and the HHS Information Security and Privacy Policy. This study implemented controls at the Federal Information Security Management Act Moderate baseline with selected additional enhancing controls wherever required. The recommendations of the Data Security Policy Principles and Framework were utilized alongside NIST-based standards and guidelines^28^. United States data security guidelines, framework, and requirements are followed internationally and thus were appropriately applied for implementation of this study in India.

### Data variables

At baseline, participants were requested to complete survey modules encompassing demographics, health history, mood and behavior, and lifestyle questionnaires (Table 1). The health history survey gathered specific inputs on COVID-19 testing, vaccination, COVID-19 infection history, and the presence of acute and long-term COVID-related symptoms. Participants’ experience with acute COVID-19 infection was captured by questions that assessed the physical impact of COVID-19 infection (e.g., hospitalization, length of recovery, and remaining symptoms). The health history survey provided overall health status and identified common chronic diseases within the cohort. The mood and behavior modules included the nine-item Patient Health Questionnaire (PHQ-9) and a seven-item General Anxiety Disorder (GAD-7) to screen for symptoms of depressed mood or anxiety, respectively^29,30^. Both PHQ-9 and GAD-7 have been validated in India^18,31^. The Pittsburgh Sleep Quality Index (PSQI) was used for the subjective assessment of sleep quality^32^. The International Physical Activity Questionnaire-Short Form (IPAQ-SF) was used to measure amounts of physical activity^33^. The lifestyle module also included questions to examine the impact of COVID-related social distancing and quarantine (e.g., loss of income/job/business or being unable to visit family members). At baseline, participants self-reported their height, weight, and blood type along with recent complete blood count, Hemoglobin A1c, and COVID (Antigen or Antibody) test results, if available. After completion of baseline measures, optional abbreviated follow-up health, mood, and lifestyle surveys were requested every four weeks through push notifications and SMS messages from the study app. At monthly follow-ups, participants were asked to record any changes in weight, updated test results, and/or symptomatic changes.

**Table 1:** Data4Life survey modules and domains for basic demographic characteristics, health history, mood, and social factors.

Survey	Domains	Sources	Baseline	Monthly
About You	Demographics	India Census questions (censusindia.gov.in)	√	
	Participant Feedback			
Health History	Health Background		√	
	Current Symptoms	Center for Disease Control website:	√	√
	COVID history	www.cdc.gov.coronavirus	√	√
	Physical Measurements		√	√
Mood	Social Connections	Berkman LF &Syme SL 1979^48^ Hughes 2004^49^	√	√
	Isolation Risks		√	√
	Depression (PHQ-9)	Kroenke K 2001^29^	√	√
	Anxiety (GAD-7)	Spitzer RL 2006^30^	√	√
Lifestyle	Activity Levels (IPAQ-SF)	Lee PH 2011^33^	√	√
	Sleep Patterns (PSQI)	Buysse DJ 1989^32^	√	√
	Alcohol/Tobacco		√	√
	Dietary Habits		√	√

Abbreviations: *PHQ-9* Patient Health Questionnaire-nine items, *GAD-7* General Anxiety Disorder-seven items, *IPAQ-SF* International Physical Activity Questionnaire-Short Form, *PSQI* Pittsburgh Sleep Quality Index.

### Data Sources and Measurements

For this preliminary report, we evaluated responses from Data4life participants enrolled from March 15, 2022, to June 13, 2022. Data was collected via self-report by completion of study modules at baseline. To date, repeated follow-up measures have not been completed and are not included in the analyses. The surveys collected demographic data (sex, age, religion, education, income, and health insurance status) from the About You module and COVID data from the Health History module (Table 1). At baseline, participants were asked to report their personal history of COVID-19 infection, COVID test, vaccinations, current symptoms, and severity, along with recent COVID-19 infection among other household members. In addition, participants completed questionnaires related to study motivation and recruitment preferences. Participants were asked to report their motivation(s) for joining the Data4Life study from a list of options: (1) to learn how to protect my health; (2) to help my family and friends; (3) to help my country; (4) to advance medical research; (5) to access latest medical advice from CDC and US National Institute of Health (NIH); and (6) other reasons (participants were asked to specify). Participants were asked to select how they were recruited into the study by selecting one or more responses from a list: (1) clinical coordinator referral; (2) family/friend referral; (3) pamphlet/newspaper; (4) newsletter/email; (5) radio; (6) search engine (e.g., Google); (7) social media; and (8) other. To gain further insights into social and cultural factors influencing participants’ preferred recruitment, the following questions were asked: (1) number of household members; (2) do you participate in social activities such as religion, charity, public service, community group, or senior center? (3) During COVID-19, did you miss getting together with family and friends and social gatherings? (4) The number of family members and friends that you feel comfortable to talk to about private matters. Questions two, three, and four were obtained from the Mood and Behavioral module. Participants’ responses to these social-related questions were introduced as binary categorical variables and used as predictors in the subsequent univariable and multivariable regression analyses to determine the impact of these social factors on preferred recruitment methods.

All data analyses were conducted using Excel and RStudio’s internal functions for regression analyses and calculation of standard statistics^34^. Key variables related to demographic information and COVID-19 status were summarized as categorical variables using frequencies and percentages. Univariable and multivariable logistic regression analyses were conducted to find independent predictive social and cultural factors for the most preferred recruitment method. Participants who did not complete the survey responses from the Mood and Behavioral module (social questions two, three, and four) were excluded from the regression analyses. The statistical significance of the univariable model was set at a *p*-value less than 0.0125 (0.05/4 to account for multiple comparisons). Clinically significant variables were evaluated using a multivariable model with age and sex added as covariates. Odds ratios (ORs) with associated confidence intervals (CI) and *p*-values were reported. To address potential sources of bias due to the collinearity between the predictor variables, we compared the ORs and *p*-values before and after removing some of the highly correlated variables.

The pilot study was designed during the early phases of the COVID pandemic, which made sample size calculation challenging due to a general lack of knowledge about PASC incidence, etiology, and presentation. Therefore, the pilot study’s sample size of 10,000 was based on preliminary estimates of the availability of COVID-positive participants in the local lab databases.

### Reporting summary

Further information on research design is available in the Nature Portfolio Reporting Summary linked to this article.

## Results

**Fig. 1:**
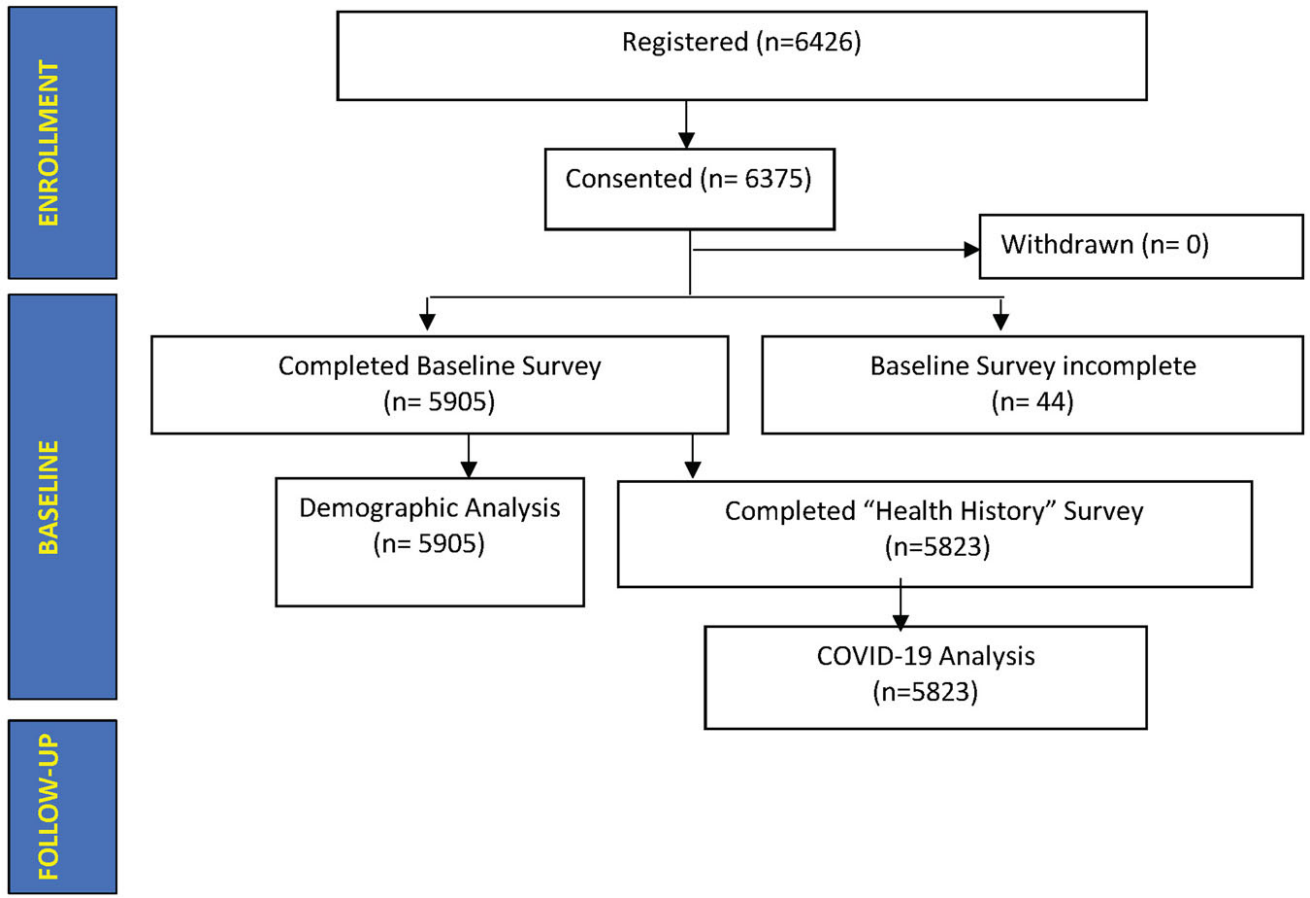
Participant baseline survey data. Data from 5905 participants who completed their baseline survey were used for demographic analysis. Totally, 5823 participants completed their Health History survey, including a basic COVID-19 questionnaire.

**Fig. 2:**
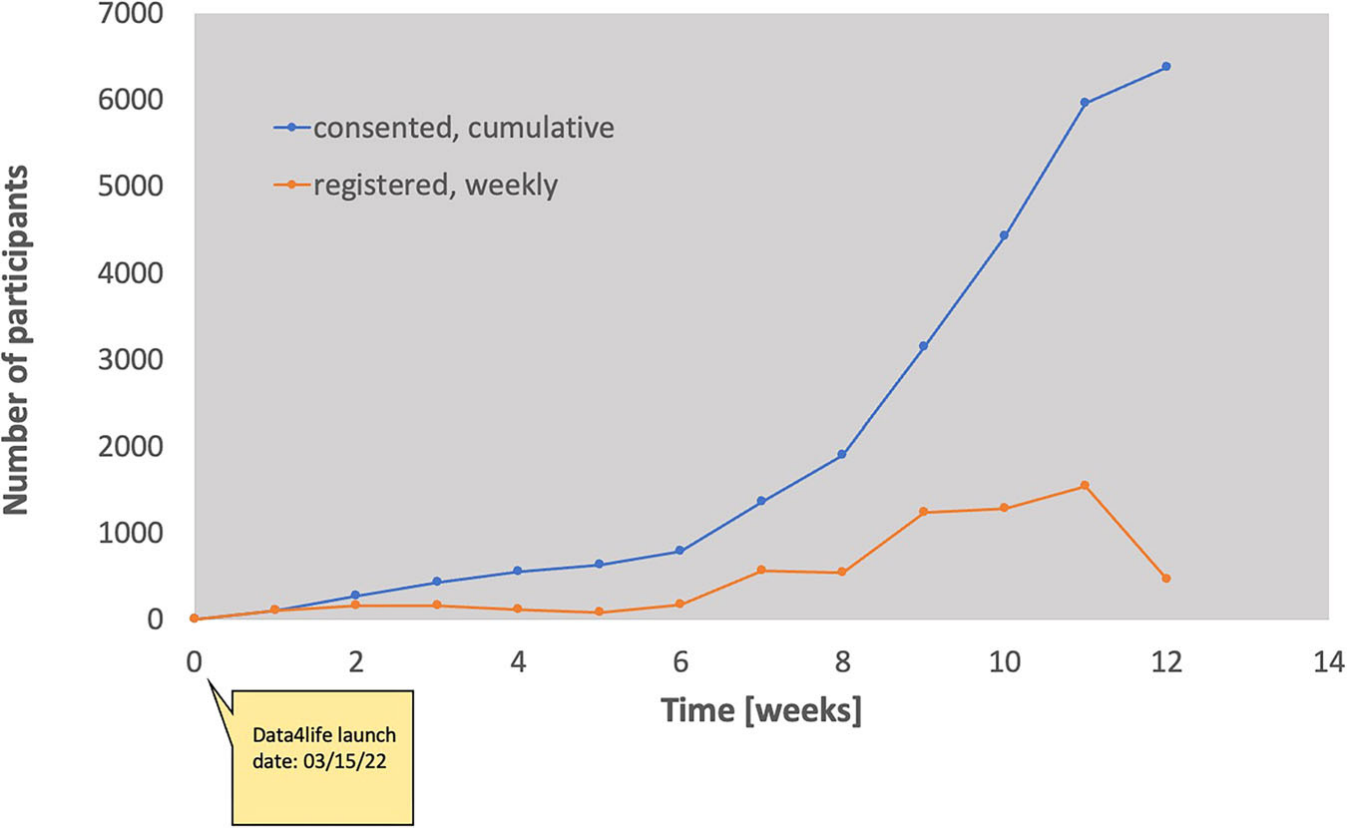
Number of registered, consented, and withdrawn participants. Weekly registrations (orange circles) and cumulative consents (blue circles) between 03-15-2022 and 06-13-2022. Out of 6426 registered individuals, 6375 participants consented.

In the first three months of recruitment, 6426 potentially eligible individuals registered for the study. As of June 13, 2022, 63.75.% (*n* = 6375) of the expected 10,000 pilot participants were enrolled, with 92.63 % (*n* = 5905) of enrolled participants completing all baseline surveys (Fig. 1). Enrollment increased by 100% between weeks 9 and 10, followed by three consecutive weeks (weeks 10–12) in which 1000+ participants were enrolled each week (Fig. 2).

**Fig. 3:**
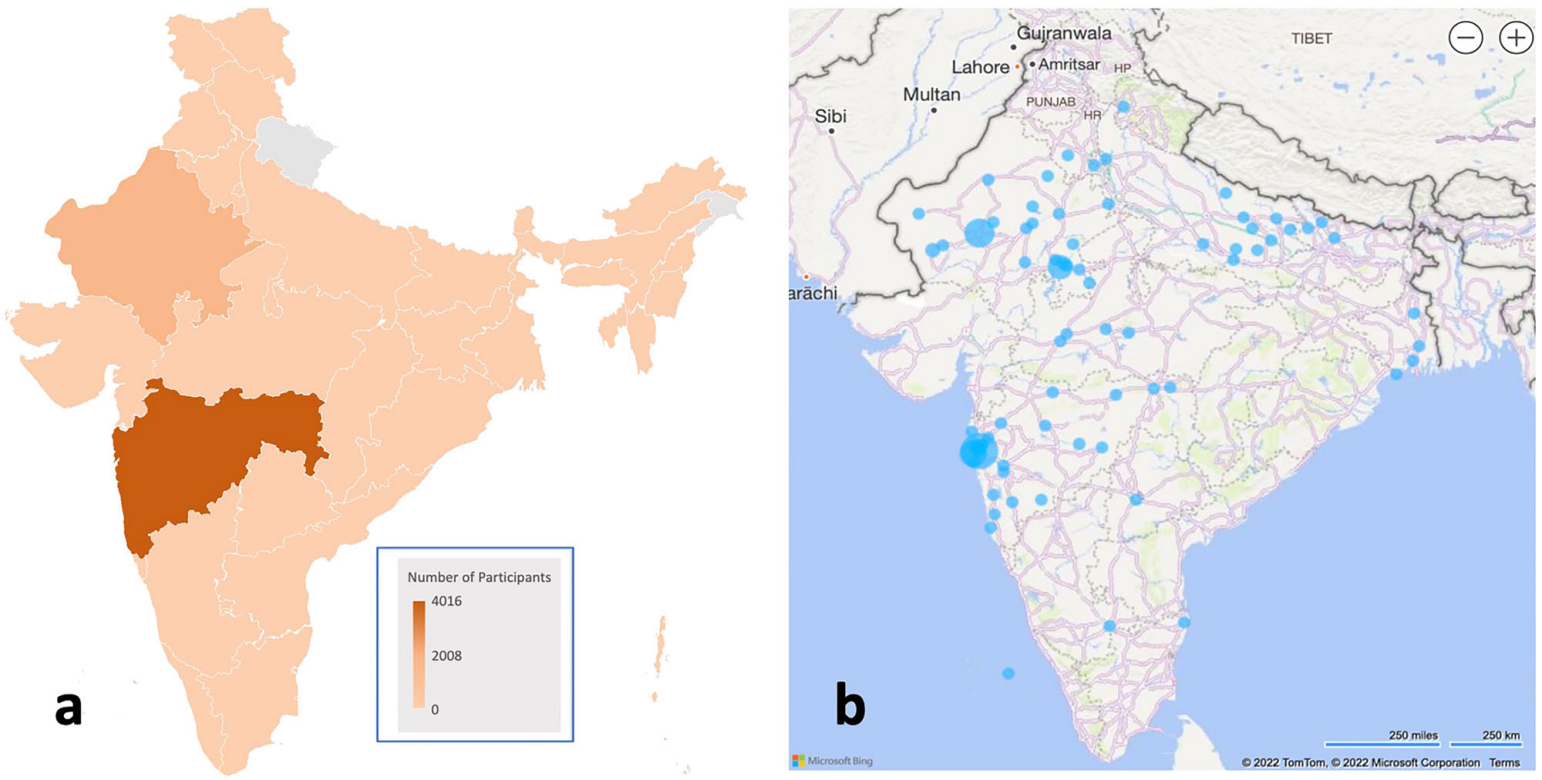
Geographic location of the participants. Location of participants by resident state (**a**) and birth city (**b**). The shades of orange in **a** represent participant densities in each state. In **b**, blue circles on India’s map indicate participant clusters within a city or metropolitan area, and the circle diameters correspond to the number of individuals in each cluster.

A total of 5905 participants who completed consent and all baseline survey modules were included in the demographic analysis. Of those participants, 74.02% were male (*n* = 4371) with mean age = 32 ± 11 years (Table 2). A household income of fewer than five lakhs/year was reported by 75.72% (*n* = 4471) of the cohort, with 51.77% being married (*n* = 3057) and approximately two-fifths of participants having a secondary school (*n* = 2756; 46.67%) education. Regarding religion, most participants identified as Hindu (*n* = 4269; 72.29%) or Muslim (*n* = 1400; 23.71%). The remaining participants (*n* = 236; 4%) were Christian, Buddhist, Jain, or identified as other. Further, 80.41% of participants (*n* = 4748) did not have health insurance, and 92.95% (*n* = 5489) reported no disability (vision, hearing, speech, mental, developmental, or other). The cohort had representation from 17 of the 28 states in India. Data showed that two-thirds of participants resided in Maharashtra (68.16%, *n* = 4025), and another 29.55% (*n* = 1745) were residents of Rajasthan (Fig. 3).

**Table 2:** Sociodemographic profile (*n* = 5905) including sex, marital status, age, education, religion, income, health insurance, and disability status.

Variable	Category	Number (N)	Frequency (%)
Sex at Birth	Male	4371	74.02
	Female	1533	25.96
	Other	1	0.02
Age (years)	18–29	3131	53.02
	30–39	1577	26.71
	40–49	726	12.29
	50–64	387	6.55
	≥65	84	1.42
Marital Status	Married	3057	51.77
	Single	2848	48.23
Education	Secondary school	2756	46.67
	Graduate	2508	42.47
	Post-graduate, Doctorate	293	4.96
	Other	348	5.89
Religion	Hindu	4269	72.29
	Muslim	1400	23.71
	Other	236	4.00
Income (annual)	<5 Lakhs	4471	75.72
	≥5 Lakhs	1434	24.28
Health insurance	Yes	1075	18.20
	No	4748	80.41
	Not responded	82	1.39
Disability	No	5489	92.96
	Yes	416	7.04

In Marital Status: Singles include widowed and divorced; In Religion: Other includes Buddhist, Christian, Sikh, Jain, and other religions.

Totally, 5823 participants were used for all remaining analyses. Currently, 91.76% (*n* = 5343) of the cohort reported testing positive for COVID-19 in the past. Most participants (*n* = 5761, 98.93%) were fully vaccinated (Table 3), and Covishield was the most received vaccine manufacturer (*n* = 5107, 87.70%). Three-fourths of individuals with positive COVID history (*n* = 3902, 73.03%) had experienced between 1 and 4 persistent symptoms of PASC, also known as long-COVID^24^. Moreover, 74.41% (*n* = 3976) of participants reported having any persistent COVID symptoms (between 1 and 8). At baseline, weakness was identified as the most common persistent symptom, followed by loss of taste or smell and respiratory issues. The distribution of reported COVID-19 diagnosis dates is presented in Supplementary Fig. 1.

**Table 3:** Participants’ (*n* = 5823) COVID-19 history, vaccination status, frequency, and type of remaining symptoms.

Variable	Category	Number	Frequency (%)
Ever tested positive for coronavirus	Yes	5343	91.76
	No	480	8.24
Tested for coronavirus in the past 4 weeks?	No, not been tested in the last 4 weeks	5432	93.29
	Yes, tested positive	70	1.20
	Yes, negative	298	5.12
	Yes, inconclusive	4	0.07
	Yes, pending	19	0.33
COVID in households in the past 4 weeks	No one tested ever	4904	84.22
	Yes, they tested positive in the past 4 weeks	46	0.79
	Yes, but they have never tested positive	206	3.54
	Yes, but it has been more than 4 weeks	667	11.45
Vaccination	Yes, Covishield	5107	87.70
	Yes, Covaxin	633	10.87
	Yes, another manufacturer	21	0.36
	No	62	1.06
Remaining symptoms	No symptoms	1367	25.58
	1–4	3902	73.03
	5–8	74	1.38
Type of symptoms	Respiratory	901	16.86
	Headache or eye-related	301	5.63
	Heart palpitation	31	0.58
	Digestion or urinary	52	0.97
	Skin	32	0.60
	Pain or numbness in the upper body	76	1.42
	Pain or numbness in the lower body	64	1.20
	Deep vein Thrombosis	4	0.07
	Lack of smell or taste	943	17.65
	Lack of appetite	224	2.36
	Hair loss	120	4.19
	Loss of sleep/insomnia	28	0.52
	General weakness	2786	52.14
	Fatigue	195	3.65
	Other symptoms	5	0.09
	No persistent effects	1356	23.38

**Fig. 4:**
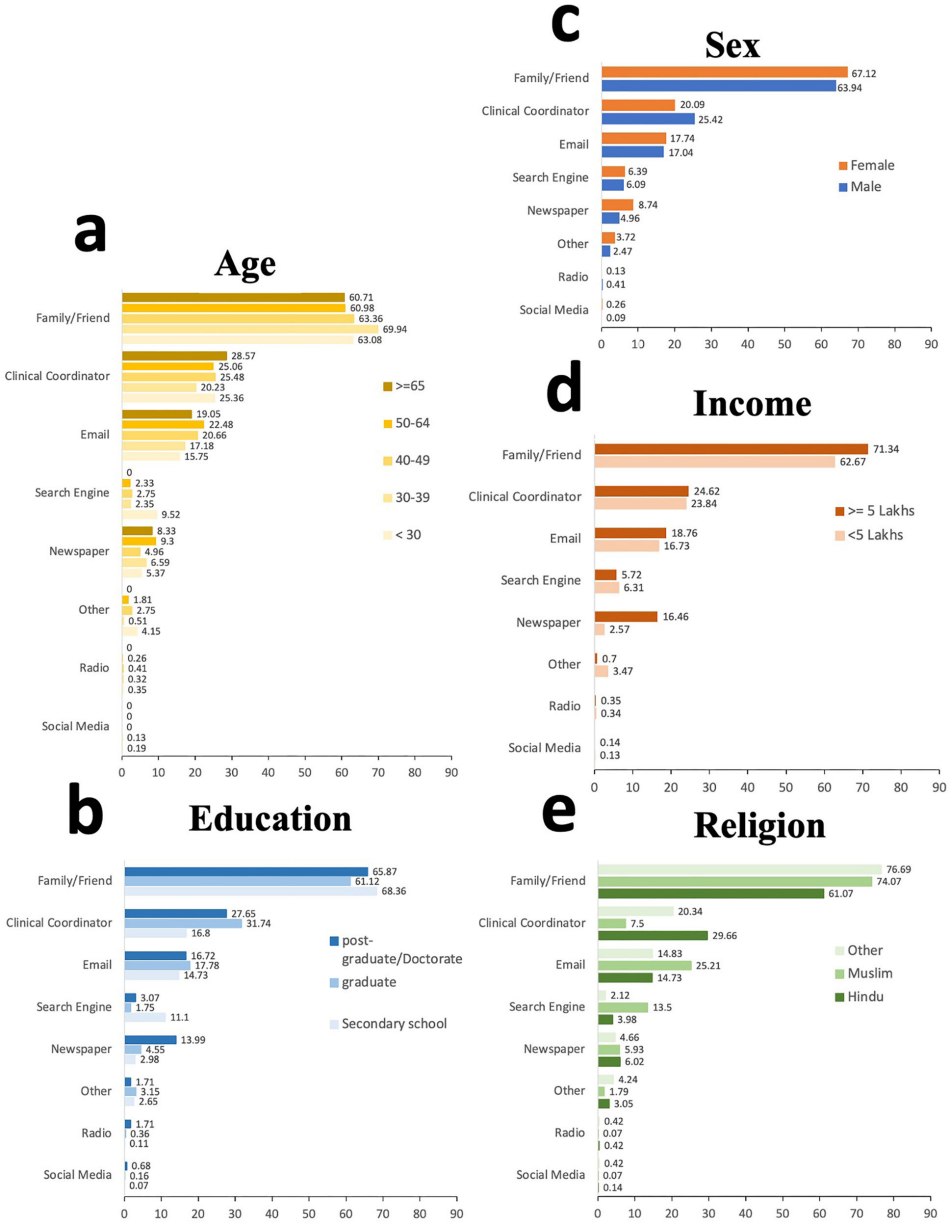
Participants by study motivation and demographic groups. Frequencies of study motivation (helping family and friends, helping the country, protecting my health, advancing research, and access to medical advice through reliable sources such as NIH) across demographic groups stratified by age (**a**), education (**b**), sex (**c**), Income (**d**), and religion (**e**). Sample size: *n* = 5905 participants were used to derive the percentages. Sample size: 5905 participants were used to derive the percentages. In **a**, the shades of yellow represent different age groups, while in **b**, different shades of blue signify educational groups. The colors orange and blue in **c** indicate the female and male sexes, respectively. In **d**, different shades of orange are used to denote income groups, and in **e**, various shades of green represent religious groups.

Frequencies of selected reasons for participation across different demographic groups were stratified by sex, age, income, education, and religion to further examine differences in motivation (Fig. 4). Helping family and friends was the most common reason for joining the study at 61.54% (*n* = 3634), followed by helping the country 50.16% (*n* = 2962) and learning how to protect health 32.58% (*n* = 1924). Access to CDC or NIH medical advice and other reasons were less commonly selected as motivating factors.

**Fig. 5:**
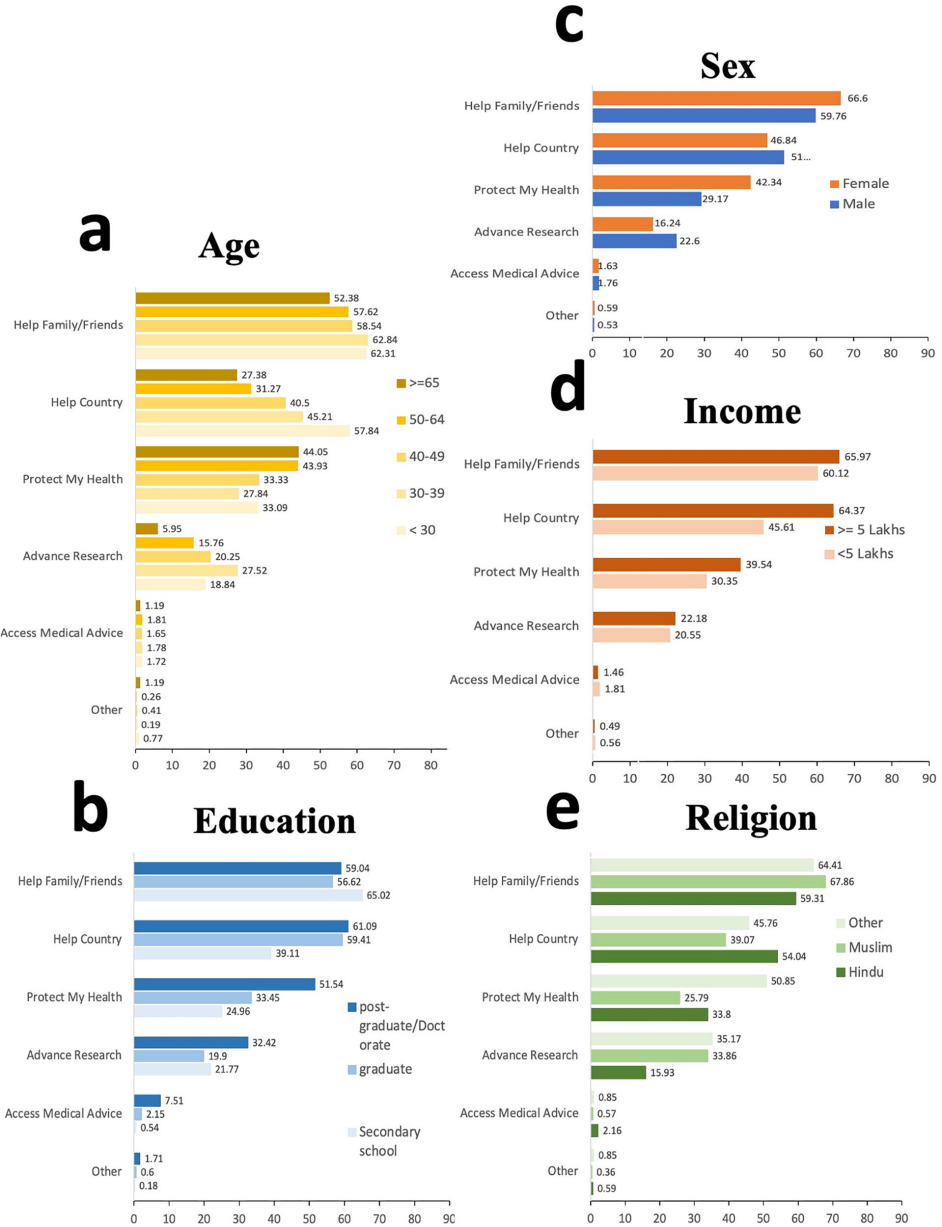
Participants by recruitment methods and demographic groups. Frequencies of recruitment methods (referral by family or friends, referral by the clinical coordinator, newsletter/email, pamphlet/newspaper, search engine, radio, social media, others) across demographic groups stratified by age (**a**), education (**b**), sex (**c**), Income (**d**), and religion (**e**). Sample size: *n* = 5905 participants were used to derive the percentages. In **a**, the shades of yellow represent different age groups, while in **b**, various shades of blue indicate educational groups. The colors orange and blue in **c** signify the female and male sexes, respectively. In **d**, different shades of orange are used to depict income groups, and in **e**, various shades of green represent religious groups.

Participants were asked to identify how they were recruited into the study. The frequency of selected recruitment methods across different groups was stratified by sex, age, income, education, and religion (Fig. 5). Friend and family referrals were the most reported recruitment method, 64.78% (*n* = 3825), followed by clinical coordinator referrals, 24.03% (*n* = 1419). Preference toward family and friend referral was observed across all demographic groups.

We further examined responses from participants’ reporting family and friend referrals as their recruitment method. We conducted univariable and multivariable logistic regression analyses to identify how various social factors were associated with selecting family and friend referrals as the recruitment method (Table 4). Ninety-six participants who had not yet completed the Mood and Behavioral module were excluded from the regression analyses. Based on the multivariable regression analysis, the likelihood of selecting family and friend referrals as the recruitment method was lower in participants who were living alone (OR = 0.49, CI: 0.41–0.58, *p* < 0.001) when compared to those who lived with others in their household. Individuals who reported they did not miss social gatherings with friends and family during the lockdown were less likely to select family/friend recruitment (OR = 0.68, CI: 0.54–0.86, *p* = 0.001) than those who missed social activities. Individuals who participated in social activities were more likely to select family and friend referrals as their recruitment method (OR = 2.08, CI: 1.73–2.52, *p* < 0.001). To address collinearity, we removed variables showing significant correlations (chi-square test with *p*-values < 0.05) from the regression model and compared the odds ratios before and after their removal. For participants who were living alone, the likelihood of selecting family/friend referrals remained low (OR: 0.51, CI: 0.44–0.59, *p* < 0.001), and individuals who participated in social activities were more likely to select family/friend referrals (OR: 2.13, CI: 1.78–2.56, *p* < 0.001).

**Table 4:** Social factors related to recruitment through family and friend referrals.

Category	Recruited through family or friend referrals		Univariate OR (95% CI, *p*-value)	Multivariate OR (95% CI, *p*-value)
Name of the categorical variable: description	No (*N* = 2080) *N* (%)	Yes (*N* = 3825) *N* (%)		
What is the number of people in your household?				
Hs1: 1 (myself)	484 (23.69)	529 (13.83)	0.52 (CI: 0.45–0.59, *p* < 0.001)	0.49 (CI:0.41–0.58, *p* < 0.001)
Hs2: 2–4 people	1002 (48.17)	2150 (56.21)	1.42 (CI: 1.28–1.59, *p* < 0.001)	1.13 (C:0.99–1.30, *p* = 0.06)
Hs3: 4+	594 (28.08)	1146 (29.96)	1.05 (CI: 0.93–1.18, *p* = 0.41)	–
Do you participate in any groups (social, work, religious self-help, charity, public service, community group, senior center)?				
S1: Yes	169 (8.13)	607 (15.87)	2.10 (CI: 1.75–2.51, (*p* < 0.001)	2.08 (CI: 1.73–2.52, *p* < 0.001)
S2: No	1856 (89.23)	3177 (83.06)	0.48 (CI: 0.40–0.57, *p* < 0.001)	–
Not responded (removed)	55 (2.64)	41 (1.07)	–	–
During COVID-19, did you miss getting together with family and friends and social gatherings?				
G1: Hardly ever	688 (33.1)	835 (21.83)	0.55 (CI: 0.49–0.62, *p* < 0.001)	0.68 (CI: 0.54–0.86, *p* = 0.001)
G2: Sometimes	1175 (56.49)	2695 (70.46)	1.79 (CI: 1.60–2.00, *p* < 0.001)	1.28 (CI: 1.03–1.59, *p* = 0.03)
G3: Often	162 (7.79)	254 (6.64)	0.83 (CI: 0.67–1.02, *p* = 0.07)	–
Not responded (removed)	55 (2.64)	41 (1.07)	–	
A number of close friends and family members that you feel comfortable to talk about private matters.				
C1: None	595 (28.61)	1566 (40.94)	1.70 (CI: 1.51–1.90, *p* < 0.001)	2.17 (CI: 1.17–4.04, *p* = 0.014)
C2: 1–2	1184 (56.92)	1935 (50.59)	0.74 (CI: 0.68–0.82, *p* < 0.001)	1.44 (CI: 0.77–2.67, *p* = 0.25)
C3: 3–5	155 (7.45)	192 (5.02)	0.64 (CI: 0.52–0.80, *p* < 0.001)	0.88 (CI: 0.46–1.69, *p* = 0.71)
C4: 6–9	72 (3.46)	64 (1.67)	0.47 (CI: 0.33–0.66, *p* < 0.001)	0.60 (CI: 0.30–1.20, *p* = 0.15)
C5: 10 or more	19 (0.91)	27 (0.71)	0.76 (CI: 0.42–1.37, *p* = 0.386)	–
Not responded (removed)	55 (2.64)	41 (1.07)	–	

Reported values include the number of participants and frequencies (%) for each category, univariate and multivariate ORs with CIs, and *p*-values. All participants (5905) across all ages between 18 and 82 years were included. Age and sex were added as covariates in the multivariate regression analyses.

Bonferroni adjusted threshold for significance is 0.0125 (0.05/4).

Abbreviations: *N* Number of participants, *OR* odds ratio, *CI* confidence interval, *P-value* probability value, Hs1–3: response categories 1–3 to the question about the number of people in household, *S1–2* response categories 1–2 to the question regarding participation in social groups, *G1–3* response categories 1–3 to the question about missing social gathering during COVID-19 pandemic, *C1–5* response categories 1–5 to the question related to the number of close friends and family members with whom the participants felt comfortable talking about private matters.

## Discussion

Data4life aimed to create a large, nationwide cohort to broadly evaluate PASC and the long-term, non-clinical effects of the COVID-19 pandemic in India. In 2020, India’s Prime Minister launched the Ayushman Bharat Digital Mission to develop an integrated digital health infrastructure. The initiation of the Data4Life Consortium supported the mission’s objective to leverage health data analytics and medical research to improve India’s health sector^35^. In just three months, the study successfully met 59.05% (*n* = 5905) of its expected 12-month recruitment goal. Further, this study demonstrated a 99.21% conversion rate of registered prospects (*n* = 6426) to fully enrolled study participants (*n* = 6375). The high conversion rate, along with the exponential increase in enrollment in weeks 10 through 12, supports the feasibility of conducting digitally enabled health research in India on a large scale. It is important to note that a local, India-based recruitment team was utilized for this study. The concentrated efforts of the local recruitment team, versus using a US-based team, may have been a strong contributing factor in the large uptick in study enrollment observed during weeks 10–12. This surge in participation may have also been influenced in part by growing COVID-related concerns with the reinstatement of international travel in India on March 27, 2022, and the emergence of the highly-transmissible COVID Omicron XE variant in India in April 2022^36^. The direct effects of COVID infection rates on study recruitment cannot be clearly determined at this time as the study is ongoing.

Data4Life was designed to capture the post-acute effects of COVID-19 in India through prospective, decentralized, digitally enabled data collection. Several evidence-based recruitment methods were employed in this study; however, preliminary findings showed that social relationships were the primary intrinsic motivating factor for participants to join the study. The most reported method of recruitment was family/friend referral. Recruitment by family and friend referral was more likely to be selected by individuals who engaged in social activities. This recruitment method was less likely to be reported amongst individuals who lived alone and those who reportedly did not miss social engagements during the lockdown period. These findings highlight the importance of social relationships and combining person-to-person recruitment with digitally enabled recruitment modalities in India. These results align with previous studies highlighting the role of family and social relationships in contemporary collectivist Indian society^37,38^. Recent studies demonstrated the necessity of face-to-face communication in social and behavioral change interventions to improve vaccination uptake and child health in rural India^39,40^. These results reflect unique characteristics within Indian social culture that may contribute to the recruitment of a large cohort using digital tools. These findings can serve as a foundation to develop culturally relevant, “best-practice” recruitment strategies that benefit from the strong sense of community and established trust in Indian culture^41^. Further evaluation will be done upon completion of the study.

This study had some limitations. One limitation of the preliminary results is that the current cohort does not adequately reflect the demographics of the general Indian population. For example, the current study cohort is 74.02% male, while India is reportedly 52% male^42^. It is estimated that 14.2% of Indians are Muslim, but Data4Life is currently 23.71% Muslim^37^. Only 2.3% of Data4Life participants reported annual salaries of 10 lakhs and higher, but this group makes up 8% of the general Indian population. Overall, our cohort represents more young males, Muslims, and individuals with higher educational attainment than seen in the general Indian population. This imbalance may be partially explained by the phasic manner in which study recruitment was initially launched in only two states. Therefore, this discrepancy may dissipate as the study continues. The high percentage of young male participants may be an unintended result of study eligibility criteria requiring participants to be literate in English. Though English is India’s second most widely spoken language, there are over one thousand dialects throughout the country. Successful recruitment of diverse populations in India will require strategies to accommodate the country’s linguistic diversity. Future study designs should include validated strategies to increase the recruitment of women, elderly adults, and individuals with various education and income levels to better represent India’s diversity. Consideration should be given to expanded recruitment efforts in states not currently represented and the inclusion of study materials in additional languages.

One of the primary objectives of this study was to gain real-time insights into our cohort’s representativeness to mitigate inherent bias associated with this method of decentralized data collection. Traditional mail-based surveys rely on well-established designs (e.g., probability-based random sampling) to build cohorts well representative of target populations. In comparison, participants in electronic cohorts are typically self-selected volunteers who may be recruited through various online and offline approaches. Low representativeness and bias are not uncommon among decentralized research studies and electronic cohorts^43^. In terms of sampling, Data4life represents a unique, technology-driven electronic cohort that combined traditional approaches, such as face-to-face conversation and clinical coordinator recruitment, with technology-driven characteristics of an e-cohort (self-elected access to the web and mobile apps). Our findings implicate the critical role of clinical coordinators and close-knit community members in successful e-cohort recruitment, reflecting the strong sense of community in India’s culture and interpersonal trust within the community. These findings may inform future strategies to mitigate unintended biases in data collection to improve representativeness by further leveraging the support of onsite communities, centers, and coordinating teams. We will seek to increase the involvement of the local coordinator teams in the future design of community campaigns targeting women, elderly adults, and individuals with lower education and income levels. These recruitment approaches may be combined with advanced data mining tools^44^ for qualitative analysis of participant characteristics and e-cohort data. For instance, advanced cluster analysis techniques can be employed to identify groups of participants who share similar demographic and clinical characteristics. This analysis enables the identification of underrepresented clusters that necessitate targeted recruitment and retention efforts. These endeavors can be facilitated through focused community campaigns aimed at engaging and retaining individuals from these specific clusters.

The high rate of COVID-positivity and vaccination in the current cohort may be considered another study limitation. 91.76% of current Data4Life participants reported testing positive for COVID-19, and 98.9% have been fully vaccinated (Table 2). By comparison, national data suggests that 88% of India had been COVID-positive, and 63.22% of India was fully vaccinated as of June 9, 2022^45^. The high positivity rate within the study was an expected result as testing COVID-positive was an eligibility criterion. However, this could be a study limitation because the exclusion of COVID-negative participants consequently excludes the collection of additional data demonstrating the non-clinical, social, economic, and lifestyle effects of COVID within the community at large. As all Indians were affected by lockdown and quarantine throughout the pandemic, the usefulness of data from COVID-negative or otherwise unaffected individuals should be considered important for comparative analyses in future studies.

India has been disproportionately affected by COVID-19. Long-term clinical consequences of COVID-19 and PASC are not fully understood, making the diagnosis and management of long-COVID challenging. It is also important to consider the non-clinical, social determinant population health effects of pandemic conditions as well. Most large-scale studies generating knowledge about the post-acute and non-clinical consequences of COVID-19 have been conducted in Europe and the US^46,47^. India’s shortage of trained workforce in health information and management, varying state regulations, lack of data governance framework, and varied data protection policies create barriers to developing digitally enabled health research cohorts^14,15^. To address this, Data4Life used a digitally-enabled longitudinal study design to build a large, observational cohort to evaluate both the clinical and non-clinical effects of PASC in India. Preliminary results show that social relationships were a key motivating factor for joining the study, which may have been critical to successful recruitment in this population. These findings may be useful in establishing effective study recruitment methods and materials for future health initiatives in India. The strong influence of social family/friend relationships in Indian culture may inadvertently lead to the development of homogenous cohorts. A rational approach should be adopted to account for these social factors and prevent sampling bias. Given the potential for COVID-19 to progress from pandemic to endemic, it is apparent that the needs of patients with PASC will continue to increase. Initial findings support the feasibility of continuing with large-scale digital health research in India. Expanding the use of digitally enabled studies may increase the inclusion of non-hospitalized COVID-19 patients, along with those with mild or asymptomatic disease diagnosed via at-home COVID-19 testing. Expansion of the Data4Life study recruitment is essential, as several groups remain underrepresented in the cohort. Future iterations may include comparative control groups of persons who have never been infected with COVID-19. Future analyses may incorporate machine learning algorithms and data-driven cluster analyses to predict patterns of long-COVID based on PASC, vaccination rates, and the variants most prevalent during different waves of the COVID-19 pandemic.

### Supplementary information

Supplementary Information

Reporting Summary

## Data Availability

The data that support the findings of this study are available from Vibrent Health, Inc. (Vibrent), but restrictions apply to the availability of these data, which were used under license for the current study and so are not publicly available. The data are, however, available from the authors upon reasonable request and with the permission of Vibrent Health, Inc. (Vibrent).
