# Supplementary material for: Development of a decentralized cohort for studying post-acute sequelae of COVID-19 in India in the Data4life Study
Source: Commun Med (Lond). 2023 Aug 25;3:117. doi: 10.1038/s43856-023-00349-y (PMC10457339; doi:10.1038/s43856-023-00349-y)
Supplement: Supplementary file 1 — Supplementary Information [file 43856_2023_349_MOESM1_ESM.pdf]

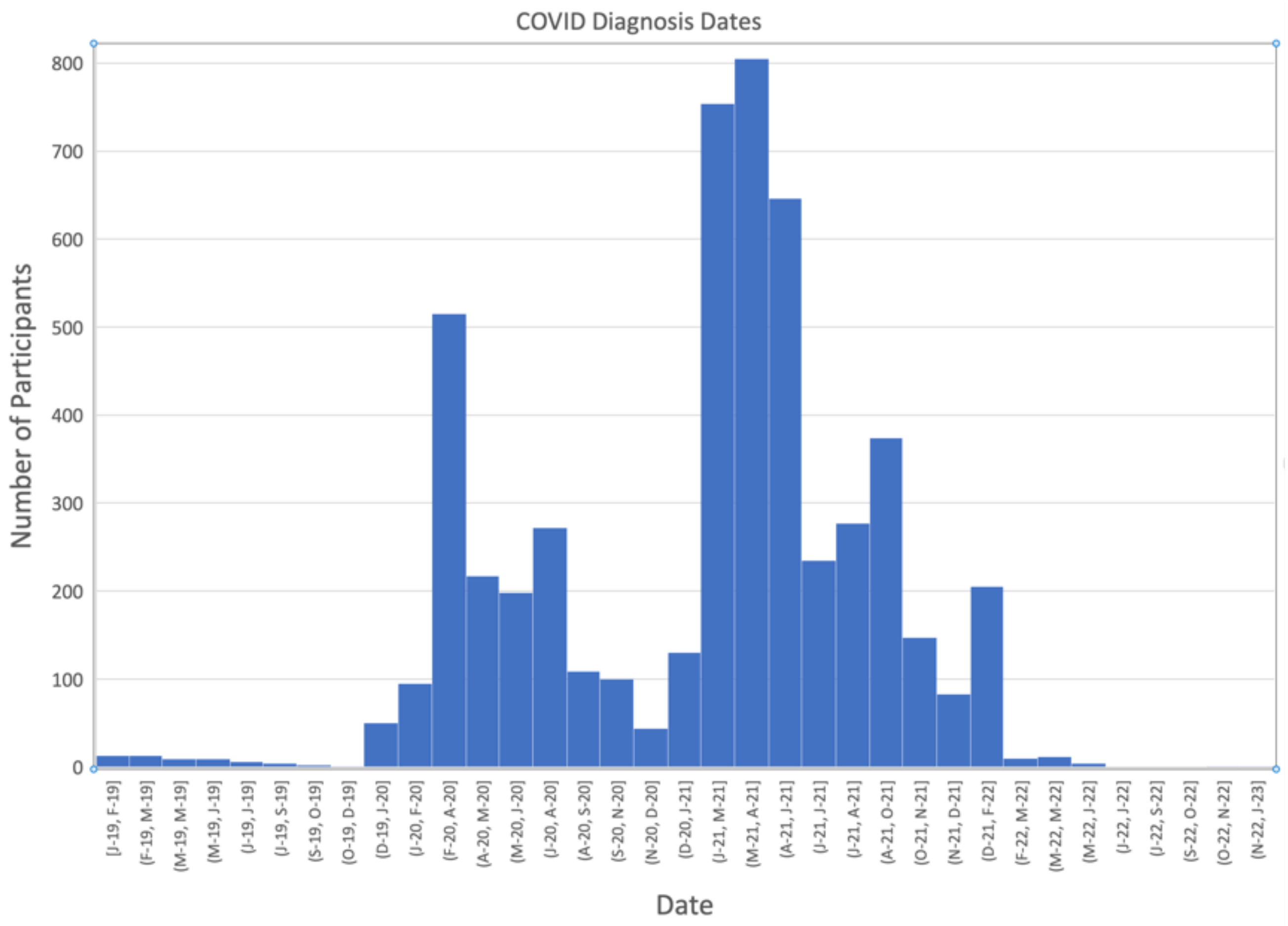

Supplementary Figure 1: Distribution of Data4life participants' COVID-19 diagnosis dates. The x axis indicates the dates and the y axis presents the number of participants.
