# Supplementary material for: Development of a decentralized cohort for studying post-acute sequelae of COVID-19 in India in the Data4life Study
Source: Commun Med (Lond). 2023 Aug 25;3:117. doi: 10.1038/s43856-023-00349-y (PMC10457339; doi:10.1038/s43856-023-00349-y)
Supplement: Supplementary file 2 — Reporting Summary [file 43856_2023_349_MOESM2_ESM.pdf]

nature portfolio

Corresponding author(s): Sepideh Shokouhi

Last updated by author(s): Aug 3, 2023

# Reporting Summary

Nature Portfolio wishes to improve the reproducibility of the work that we publish. This form provides structure for consistency and transparency in reporting. For further information on Nature Portfolio policies, see our Editorial Policies and the Editorial Policy Checklist.

## Statistics

For all statistical analyses, confirm that the following items are present in the figure legend, table legend, main text, or Methods section.

| n/a | Confirmed | |
|---|---|---|
| ☐ | ☒ | The exact sample size (*n*) for each experimental group/condition, given as a discrete number and unit of measurement |
| ☐ | ☒ | A statement on whether measurements were taken from distinct samples or whether the same sample was measured repeatedly |
| ☐ | ☒ | The statistical test(s) used AND whether they are one- or two-sided<br>*Only common tests should be described solely by name; describe more complex techniques in the Methods section.* |
| ☐ | ☒ | A description of all covariates tested |
| ☐ | ☒ | A description of any assumptions or corrections, such as tests of normality and adjustment for multiple comparisons |
| ☐ | ☒ | A full description of the statistical parameters including central tendency (e.g. means) or other basic estimates (e.g. regression coefficient) AND variation (e.g. standard deviation) or associated estimates of uncertainty (e.g. confidence intervals) |
| ☒ | ☐ | For null hypothesis testing, the test statistic (e.g. $F$, $t$, $r$) with confidence intervals, effect sizes, degrees of freedom and $P$ value noted<br>*Give P values as exact values whenever suitable.* |
| ☒ | ☐ | For Bayesian analysis, information on the choice of priors and Markov chain Monte Carlo settings |
| ☒ | ☐ | For hierarchical and complex designs, identification of the appropriate level for tests and full reporting of outcomes |
| ☒ | ☐ | Estimates of effect sizes (e.g. Cohen's *d*, Pearson's *r*), indicating how they were calculated |

*Our web collection on statistics for biologists contains articles on many of the points above.*

## Software and code

Policy information about availability of computer code

| | |
|---|---|
| Data collection | Vibrent Health digital platform was used for data collection |
| Data analysis | RStudio console and Excel were used for data analysis |

For manuscripts utilizing custom algorithms or software that are central to the research but not yet described in published literature, software must be made available to editors and reviewers. We strongly encourage code deposition in a community repository (e.g. GitHub). See the Nature Portfolio guidelines for submitting code & software for further information.

## Data

Policy information about availability of data

All manuscripts must include a data availability statement. This statement should provide the following information, where applicable:

- Accession codes, unique identifiers, or web links for publicly available datasets
- A description of any restrictions on data availability
- For clinical datasets or third party data, please ensure that the statement adheres to our policy

The data that support the findings of this study are available from Vibrent Health, Inc (Vibrent), but restrictions apply to the availability of these data, which were used under license for the current study and so are not publicly available. The data are, however, available from the authors upon reasonable request and with the permission of Vibrent Health, Inc (Vibrent).

## Human research participants

Policy information about studies involving human research participants and Sex and Gender in Research.

| | |
|---|---|
| Reporting on sex and gender | No participant was excluded from this study based on their sex or gender. The findings of this study apply to both sexes. Sex was added as covariate in the data analysis. Sex was determined based on self-reporting. This study included 4,371 male participants and 1,533 female participants. |
| Population characteristics | A total of 5,905 participants completed consent and all baseline survey modules. Currently enrolled participants are 74.02% male (n=4,371) with mean age=32±11 years. A household income of less than five lakhs/year was reported by 75.72% (n= 4,471) of the cohort, with 51.77% being married (n=3,057) and approximately two-fifths of participants having a secondary school (n= 2,756; 46.67%) education. Regarding religion, most participants identified as Hindu (n= 4,269; 72.29%) followed by Muslims (n= 1,400; 23.71%). The remaining participants (n= 236; 4%) were Christians, Buddhists, Sikhs, Jains, and individuals who identified as other. Further, 80.4% of participants (n = 4,748) reported having no health insurance, and 92.95% (n = 5,489) reported having no disability (vision, hearing, speech, mental, developmental, or other). The cohort currently has representation from 17 of the 28 states in India. Data show that two-thirds of participants reside in Maharashtra (68.16%, n =4,025), and another 29.75% (n = 1,745) are residents of Rajasthan. |
| Recruitment | Eligible participants were identified in databases of over 20,000 patients who previously tested COVID-positive at an affiliated hospital or laboratory. Clinical coordinators contacted eligible individuals and members of their households by email or phone call. Data4Life participants had the option to invite family members and friends to join the study. Social media and email campaigns were not used for active recruitment; however, enrolled participants were able to send email invitations within their social network. Furthermore, as the pandemic progressed, increased COVID testing resulted in increased promotion of the Data4life study among friends, families, and neighbors. |
| Ethics oversight | Royal Pune Independent Ethics committee in India |

Note that full information on the approval of the study protocol must also be provided in the manuscript.

# Field-specific reporting

Please select the one below that is the best fit for your research. If you are not sure, read the appropriate sections before making your selection.

☒ Life sciences ☐ Behavioural & social sciences ☐ Ecological, evolutionary & environmental sciences

For a reference copy of the document with all sections, see nature.com/documents/nr-reporting-summary-flat.pdf

# Life sciences study design

All studies must disclose on these points even when the disclosure is negative.

| | |
|---|---|
| Sample size | The design of this pilot study was completed during the early phases of the COVID pandemic, making sample size calculation challenging due to general lack of knowledge about PASC incidence, etiology, and presentation. Therefore, the pilot study's sample size of 10,000 was based on preliminary estimates on the availability of COVID-positive participants in the lab databases. |
| Data exclusions | We excluded data from incomplete baseline survey responses. |
| Replication | This is a observational pilot study. Replication was not applicable. |
| Randomization | N/A |
| Blinding | N/A |

# Reporting for specific materials, systems and methods

We require information from authors about some types of materials, experimental systems and methods used in many studies. Here, indicate whether each material, system or method listed is relevant to your study. If you are not sure if a list item applies to your research, read the appropriate section before selecting a response.

## Materials & experimental systems

| n/a | Involved in the study |
|---|---|
| ☒ | ☐ Antibodies |
| ☒ | ☐ Eukaryotic cell lines |
| ☒ | ☐ Palaeontology and archaeology |
| ☒ | ☐ Animals and other organisms |
| ☒ | ☐ Clinical data |
| ☒ | ☐ Dual use research of concern |

## Methods

| n/a | Involved in the study |
|---|---|
| ☒ | ☐ ChIP-seq |
| ☒ | ☐ Flow cytometry |
| ☒ | ☐ MRI-based neuroimaging |
